# What Each Clinical Anatomist Has to Know about Left Renal Vein Entrapment Syndrome (Nutcracker Syndrome): A Review of the Most Important Findings

**DOI:** 10.1155/2017/1746570

**Published:** 2017-12-11

**Authors:** Krzysztof Orczyk, Grzegorz Wysiadecki, Agata Majos, Ludomir Stefańczyk, Mirosław Topol, Michał Polguj

**Affiliations:** ^1^Department of Angiology, Medical University of Łódź, Narutowicza 60, 90-136 Łódź, Poland; ^2^Department of Normal and Clinical Anatomy, Medical University of Łódź, Narutowicza 60, 90-136 Łódź, Poland; ^3^Department of Radiological and Isotopic Diagnosis and Therapy, Medical University of Łódź, ul. Żeromskiego 113, 90-549 Łódź, Poland; ^4^Department of Radiology, Medical University of Łódź, Kopcińskiego 22, 90-153 Łódź, Poland

## Abstract

Nutcracker syndrome (NCS) is the most common term for compression of the left renal vein between the superior mesenteric artery and the abdominal aorta. The development of NCS is associated with the formation of the left renal vein (LRV) from the aortic collar during the sixth to eighth week of gestation and abnormal angulation of the superior mesenteric artery from the aorta. Collateralization of venous circulation is the most significant effect of NCS. It includes mainly the left gonadal vein and the communicating lumbar vein. Undiagnosed NCS may affect retroperitoneal surgery and other radiological and vascular procedures. The clinical symptoms of NCS may generally be described as renal presentation when symptoms like haematuria, left flank pain, and proteinuria occur, but urologic presentation is also possible. Radiological methods of confirming NCS include Doppler ultrasonography as a primary test, retrograde venography, which can measure the renocaval pressure gradient, computed tomography angiography, which is faster and less traumatic, intravascular ultrasound, and magnetic resonance angiography. Treatment can be conservative or surgical, depending on the severity of symptoms and degree of LRV occlusion. Nutcracker syndrome is worth considering especially in differential diagnosis of haematuria of unknown origin.

## 1. Introduction

The entrapment of the left renal vein (LRV) between the abdominal aorta (Ao) and superior mesenteric artery (SMA) is commonly referred to as nutcracker syndrome (NCS) [1, 2]. This compression often results in haematuria, which has recently attracted increasing clinical attention. However, NCS can only be diagnosed when LRV compression produces symptoms, the most frequent ones being left flank pain, varicocele, proteinuria, and anaemia [3]. As these symptoms are often presented by patients in Primary Health Care and are not specific, knowledge of NCS and its causes is essential for doctors of all specialties. It is a particularly important issue for radiologists and vascular surgeons, as the diagnosis of NCS may also affect renal/adrenal venography, venous sampling, and the treatment of thromboembolic diseases [4, 5].

The nutcracker phenomenon (NCP) should be defined separately, as the configuration of the vessels results in the compression of the left renal vein and its dilatation after narrowing in asymptomatic patients. It is frequently an incidental finding during medical imaging performed due to other causes. As the majority of NCP cases remains asymptomatic [6] and, hence, undiagnosed or may only be discovered incidentally, it is difficult to assess the real frequency of the disease. Buschi et al. [6] observed a distended LRV in 72% of healthy individuals and hypothesized that NCP may be prevalent. Therefore it is still unclear why this configuration of vessels produces symptoms only in a small part of population.

Literature data suggests that NCS is slightly more prevalent in women [7], although other studies indicate no difference in gender [3]. It is usually diagnosed in the second and third decades of life and tends to be diagnosed earlier in men [3].

The systematic analysis of papers available in MEDLINE via PUBMED, EBSCO, and SCOPUS has been done. Different sources of data were compared and summary of their analysis is presented. The study was focused on the embryology and pathophysiology of the disease, as well as its clinical image, diagnostic process, and therapeutic methods in order to clarify the clinical significance of NCS.

## 2. Embryology

The inferior vena cava (IVC) regularly develops according to a complex process which involves three pairs of fetal veins: the posterior cardinal veins, subcardinal veins, and supracardinal veins [5]. The development of the IVC begins in the fourth week of gestation with the domination of separate posterior cardinal veins. In the sixth week, a pair of subcardinal veins appear. They are connected through a midline anastomosis and empty into the posterior cardinal veins. In the seventh week, supracardinal veins arise from the cranial end of the posterior cardinal veins and then descend to the subcardinal veins. Altogether, they form a central network of anastomoses referred to as the aortic collar [8].

In the eighth week of gestation, these vessels form the four segments of the final IVC: the infrarenal, renal, suprarenal, and hepatic segments [5]. The aortic collar is crucial for development of the renal veins. Typically, the posterior part of the described anastomotic set degenerates, whereas the anterior portion becomes the left renal vein (LRV). A circumaortic LRV is observed when the posterior segment of aortic collar fails to regress. Regression of the anterior instead of the posterior part leads to the retroaortic position of the LRV [8].

When it comes to the arterial system, the paired ventral segmental arteries branch from the paired dorsal aortae and they approach each other in the mesentery. The 10th, 13th, and 21st pair of these vessels fuse in the midline and form, respectively, the celiac trunk, superior mesenteric artery (SMA), and inferior mesenteric artery (IMA) [9].

## 3. Definition and Types of NCS

The main radiological diagnostic criterion for NCS is defined by a renocaval pressure gradient measured during retrograde venography. A gradient of <1 mmHg is considered to be normal [10]. The pressure gradient between the LRV and IVC should be >2 mmHg [11] or >3 mmHg [12] to confirm the diagnosis of NCS.

Another radiological criterion postulated as confirmatory evidence of NCS [13, 14] is detection of diversified collateral veins around the LRV on Doppler ultrasonography. An increase in blood pressure in the LRV caused by NCS is the underlying mechanism resulting in collateralization [15]. According to Grimm et al. [13], the main collateral pathways [16]: left gonadal vein and the communicating lumbar vein, were dilated in 16% and 24% of NCS patients, respectively. Venous hypertension sustained by collaterals can rupture the thin-walled septum between the small veins and the collecting system in the renal fornix [17]. These abnormal communication channels are responsible for haematuria, which is the most common clinical finding of NCS [11]. On the contrary, a well-developed collateral circulation may decrease LRV hypertension and result in absence of the renal symptoms of NCS [18].

**Figure 1:**
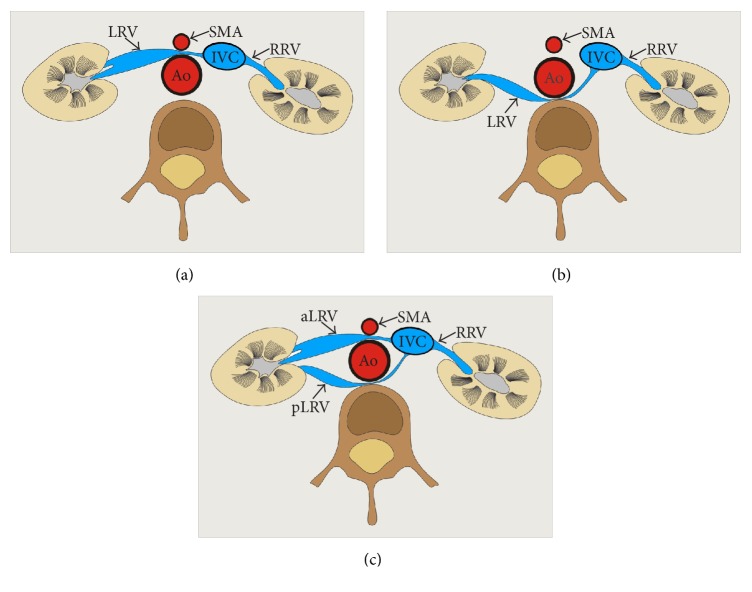
Schematic arrangements of the main types of nutcracker syndrome. (a) Anterior type, (b) posterior type (retroaortic left renal vein), and (c) posterior type (circumaortic left renal vein). Ao: abdominal aorta, IVC: inferior vena cava, aLRV: anterior left renal vein, pLRV: posterior left renal vein, and SMA: superior mesenteric artery.

**Figure 2:**
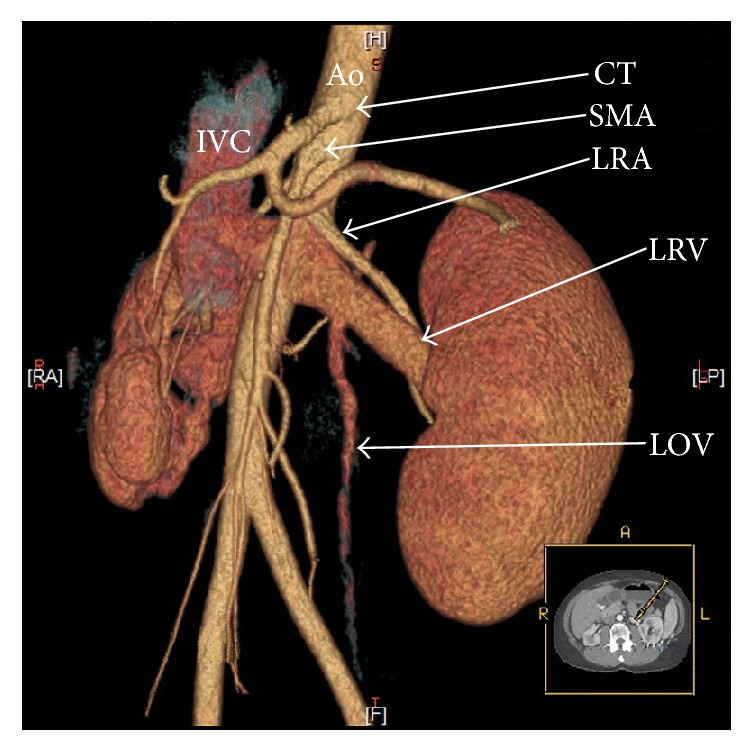
Three-dimensional computed tomography reconstruction of the abdominal arteries (CT-64-row MDCT scanner, Light-Speed VCT, GE, Waukesha, Wisconsin, US). Ao: abdominal aorta, CT: celiac trunk, IVC: inferior vena cava, LOV: left ovarian vein, LRA: left renal artery, LRV: left renal vein, and SMA: superior mesenteric artery.

**Figure 3:**
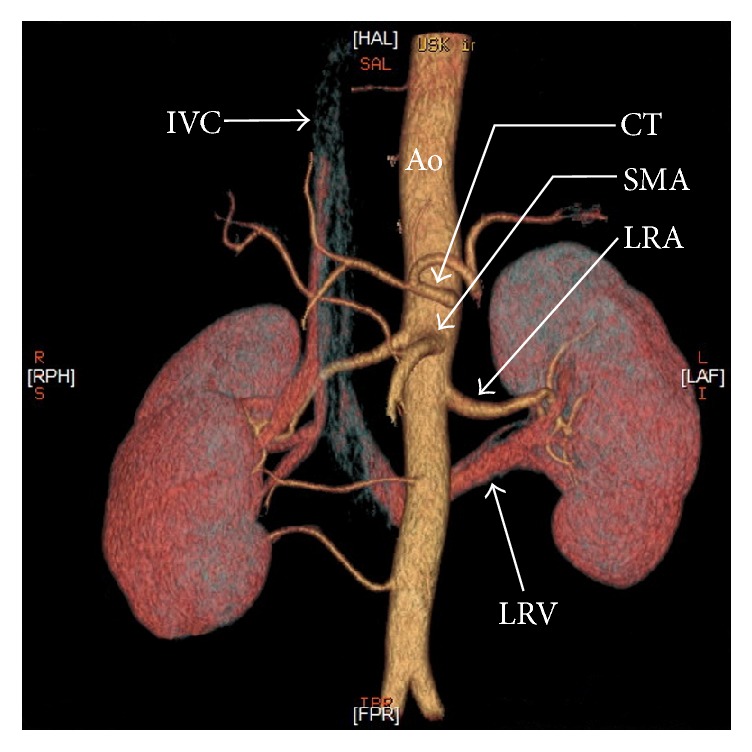
Three-dimensional computed tomography reconstruction of the abdominal arteries (CT-64-row MDCT scanner, Light-Speed VCT, GE, Waukesha, Wisconsin, US). Ao: abdominal aorta, CT: celiac trunk, IVC: inferior vena cava, LRA: left renal artery, LRV: left renal vein (retroaortic), and SMA: superior mesenteric artery.

**Figure 4:**
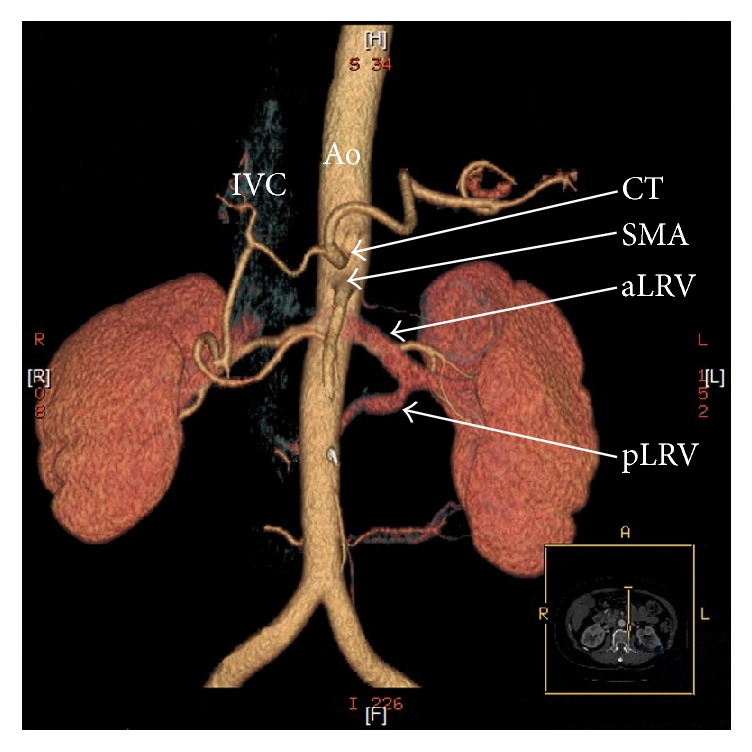
Three-dimensional computed tomography reconstruction of the abdominal arteries (CT-64-row MDCT scanner, Light-Speed VCT, GE, Waukesha, Wisconsin, US). Ao: abdominal aorta, CT: celiac trunk, IVC: inferior vena cava, aLRV: anterior left renal vein, pLRV: posterior left renal vein, and SMA: superior mesenteric artery.

Generally, there are two main types of nutcracker syndrome, described as anterior and posterior NCS [11]. In anterior NCS, the LRV is compressed between the abdominal aorta and superior mesenteric artery (Figures 1(a) and 2). The second (posterior) type results in the narrowing of the LRV in its retroaortic (Figures 1(b) and 3) or circumaortic (Figures 1(c) and 4) position: compression between the aorta and the vertebral column. Although the majority of NCS cases can be labeled as anterior NCS, the importance of the posterior variant should not be underrated. The incidence of the retro/circumaortic position of the LRV varies from 0.1% to 3.2% [8]. A retro- or circumaortic LRV, especially when not identified, is a significant risk factor of hemorrhage during aortic or retroperitoneal surgery [5, 8].

However, there are also several subtypes of NCS. Shah et al. [19] found left renal vein duplication with the retroaortic branch trapped between the vertebral column and the aorta at the level of the aortic bifurcation [19]. Polguj et al. [20] described a case when the left renal vein was compressed as it passed between the superior mesenteric artery and the right renal artery. Nakazawa et al. [21] noted compression of the left renal vein by dilated left-sided inferior vena cava. Other rare variants of NCS include right-sided NCS, which may be induced by pregnancy as a factor determining the compression of the right renal vein and the IVC [22] or by other anatomical variants like left-sided IVC [23].

Nonvascular impingement may be caused by pancreatic neoplasm, para-aortic lymphadenopathy, retroperitoneal tumor, excessive fibrolymphatic tissue between the SMA and the aorta, left renal ptosis, lordosis, or decreased retroperitoneal and mesenteric fat tissue [11]. NCS may also coincide with superior mesenteric artery syndrome, which affects the duodenum and is also provoked by the abnormal angulation of the SMA [24]. An unusual case of NCS due to acute aortic dissection has been also described [25]. The presence of the liver and pancreas at the level of the LRV has frequently been observed in NCS and was found to be an independent factor for NCS [26].

The main theories on the origin of NCS include the presence of (1) an excessively acute angle between the SMA and the abdominal aorta [15], (2) an abnormally high course of the LRV [15], and (3) retro- or circumaortic variants of the LRV [8].

The SMA usually arises from the aorta at the L1 level [11] at an angle of 90 ± 10° [27] and it courses in the central direction for 4-5 mm before turning inferiorly in an inverted J shape [28]. Such configuration may be present in up to 68% of cases [29]. The angle between the SMA and the aorta is more acute in NCS patients [30]. It frequently measures less than 35° [31] in NCS patients compared to 38–56° [32] or 51 ± 25° [11] in healthy individuals.

## 4. Clinical Features

The following clinical symptoms were present in patients with NCS [3]: haematuria in 78.57%, left flank pain in 38.39%, varicocele in 35.71% of males, proteinuria in 30.36%, and anaemia in 13.39%. The three typical symptoms (haematuria, left flank pain, and varicocele) are induced by a backward venous renal hypertension [5] and are strongly related to the development of collateral circulation. Other symptoms and concomitant diseases reported in individual cases include abdominal pain, headache, emotional disturbances, left leg varicosis, scrotal discomfort, tachycardia, left loin pain, left iliac fossa pain, abdominal aortic aneurysm, stomach ache, anxiety, chronic fatigue, syncope, fever, orthostatic intolerance, dysmenorrhea, dysuria, and dyspareunia [3, 13].

Hwang et al. [33] observed that nutcracker syndrome might be closely related to orthostatic proteinuria in children. They performed left renal venography and pressure tracing in 23 children with orthostatic proteinuria, which showed 12 cases (52%) of typical nutcracker syndrome.

Gulleroglu et al. [11] described two types of clinical image of NCS based on a classification of the aforementioned symptoms: (1) haematuria, proteinuria, and left flank pain were labeled as a* renal presentation*, and (2) the remaining symptoms (varicocele, abdominal pain, dyspareunia, dysmenorrhea, fatigue, and orthostatic intolerance) were grouped as a* urologic presentation*.

Some NCS patients may have been misdiagnosed with glomerular nephritis based on the presence of the renal presentation of NCS symptoms [32]. According to one hypothesis, proteinuria in NCS patients may be predisposed by a subtle subclinical immune injury to the glomerulus [34]. Cuéllar i Calàbria et al. [18] observed a high percentage of *γ*-globulinuria in NCS patients with orthostatic proteinuria; therefore they postulated it as a valuable marker.

Patients with NCS are usually tall and thin [35], which corresponds with the more acute angle between the SMA and the abdominal aorta in these individuals. Therefore, the increase in body mass index during childhood might improve hemodynamics in the LRV. Nutcracker syndrome may be also present in children and is believed to be an important cause of pediatric varicocele [36]. In isolated cases, nontreated, misdiagnosed NCS may produce a solitary LRV thrombosis as a severe complication [37].

## 5. Radiological Diagnostic Methods

As a patient presents with one of the typical symptoms, NCS diagnosis may be verified by medical imaging techniques. The following methods are utilized: Doppler ultrasonography, retrograde venography, intravascular ultrasound (IVUS), computed tomography angiography (CTA), and magnetic resonance angiography (MRA) [38].

Doppler ultrasonography is considered to be the most efficient initial diagnostic test. The LRV diameter and peak velocity (PV) at the level of the renal hilum and the level of the LRV course between the SMA and the aorta should be compared. If the diameter and PV ratios are >5, it is a likely indicator of NCS [39] with a sensitivity of 78% and specificity of 100% [14]. It is difficult to obtain these measurements in the supine position due to the artificial compression of the LRV caused by the transducer, which makes it virtually impossible to calculate the diameter ratio [40]. It is easier to get such values in a semisitting position [41]; however, as it is not a regular procedure, the ultrasonographer has to consider the presence of NCS before the examination. Standard CT is also insufficient to validate diameter ratios as LRV dilatation may be a normal variant [15].

The diagnosis of nutcracker syndrome is still unclear; it may be confirmed by phlebography and measurement of the venous pressure gradient between left renal vein and inferior vena cava or by intravascular ultrasound. These both procedures (phlebography and intravascular ultrasound) remain the “gold standard” during the diagnosis of nutcracker syndrome [29, 42, 43]. Intravascular ultrasound has a higher specificity of 90% compared with 62% with phlebography [42, 43]. Gill et al. [44] compared luminal diameter measurements obtained via digital subtraction angiography and intravascular ultrasound during a variety of pediatric endovascular procedures. According to their investigation in venous compression syndromes, like nutcracker syndrome intravascular ultrasonography might provide a more accurate representation of vessel compression and diameter than digital subtraction angiography.

Computed tomography angiography (CTA) has recently been gaining importance also as a gold standard in the diagnostic process of NCS. It provides a noninvasive evaluation of renal vasculature, avoiding the risks of retrograde venography, including vessel injury, pseudoaneurysm formation, and renal injury [45]. Moreover, CTA allows more detailed evaluation of the LRV and retroperitoneal anatomy [5], as well as the collateral veins, through three-dimensional reconstructive images. Another benefit of CTA is that it is a faster examination than retrograde venography or MRA [45]. The highest diagnostic accuracy observed in axial CT images is “the beak sign” (severe form of narrowing of the LRV at the aortomesenteric portion), the LRV diameter, and angle between the superior mesenteric artery and aorta <41° [42, 43]. However, MRA provides an excellent morphological definition, and the multiplanar imaging better delineates soft tissue anatomy in the region of the compression and is radiation free [29, 42]. Nevertheless, CTA has a greater potential as a gold standard due to its better accessibility.

## 6. Treatment

The therapeutic decision in NCS patients is controversial. Conservative treatment in mild LRV stenosis with no backflow remains a dominant view, especially in children. The degree and stage of NCS and the possibility of spontaneous reduction of LRV compression during childhood should be considered before surgical treatment [46]. Spontaneous remission of persistent severe haematuria in an adolescent with NCS as the consequence of increase height was also observed [47]. Also development of a visceral fat changes the anatomical relation between vascular structures and may increase compression of LRV [46, 47].

The presence of severe LRV occlusion with well-developed collateral veins and complete clinical image is considered a suitable indication for surgical treatment. Typical open approaches, like LRV transposition, mesoaortic transposition, nephropexy, renal decapsulation, and renal autotransplantation [18, 44], are effective but invasive.

Less traumatic alternative interventions should be considered as they correspond with the general idea of the minimally invasive management of NCS. These procedures include [48] transluminal balloon angioplasty, gonado-caval bypass, laparoscopic LRV transposition, and endovascular stenting of the LRV. The last one, despite its low invasiveness and potential to become a gold standard, may produce such complications as venous thrombosis, stent migration, stent protrusion into IVC, and in-stent restenosis [48]. The outcomes of laparoscopic procedures reported in the literature are comparable with those of open procedures [42, 49]. However, surgery should be considered for gross haematuria (especially if recurrent) or for severe symptoms including anaemia, left flank, or abdominal pain, impairment of renal function including persistent orthostatic proteinuria and varicocele formation, and for ineffective conservative measures after 24 months in patients aged less than 18 years and after 6 months in adults [35, 42, 50, 51].

The natural history of nutcracker syndrome is far from clear and it remains an underdiagnosed condition because of its nonspecific presentation [42]. However, without treatment nutcracker syndrome can predispose to left renal vein thrombosis [52] and kidney damage [42]. In cases of nutcracker syndrome with haematuria, it can lead to anaemia requiring blood transfusion [42, 53].

## 7. Conclusion

Left renal vein entrapment syndrome, known clinically as nutcracker syndrome, is an uncommon constellation of vessels resulting in increased blood pressure before compression. It may form collateral vascularization, resulting in diverse clinical symptoms. As patients with NCS are likely to be misdiagnosed, the presence of this uncommon pathology should be considered in patients, especially those with haematuria of unknown origin.

## Abbreviations

Ao: Abdominal aorta
CTA: Computed tomography angiography
IMA: Inferior mesenteric artery
IVC: Inferior vena cava
LRV: Left renal vein
MRA: Magnetic resonance angiography
NCP: Nutcracker phenomenon
NCS: Nutcracker syndrome
SMA: Superior mesenteric artery.
